# Hearing interventions to prevent dementia

**DOI:** 10.1007/s00106-019-0617-7

**Published:** 2019-02-14

**Authors:** P. Dawes

**Affiliations:** 1 Manchester Centre for Audiology and Deafness (ManCAD), School of Health Sciences, Faculty of Biological, Medical and Health Sciences, University of Manchester, Oxford Rd, M13 9PL Manchester, UK; 2 Cathie Marsh Institute for Social Research, University of Manchester, Manchester, UK

**Keywords:** Hearing loss, Cognitive dysfunction, Hearing aids, Cochlear implants, Outcome assessment, Hörverlust, Kognitive Funktionsstörung, Hörgeräte, Cochleaimplantate, Ergebnisbeurteilung

## Abstract

Hearing loss is a marker of risk for cognitive decline and dementia. Controlled hearing intervention studies of long-term cognitive outcomes are challenging, and thus the evidence for the impact of hearing interventions is primarily from observational studies and will likely continue to be from studies other than randomised controlled trials. Seven studies of hearing interventions with cognitive outcomes assessed over longer than 3 years are reviewed. Most were of low-to-moderate quality. One cochlear implant study had indeterminate findings. Of six hearing aid studies, three reported a positive impact of hearing aid use while three reported no impact of hearing aid use on cognitive decline or incident cognitive impairment. Further studies are required to elucidate the benefit of hearing interventions on long-term cognitive outcomes. Research should include objectively ascertained hearing data, theoretically motivated cognitive outcomes including dementia subtypes, characterisation, and control for confounds and application of advanced statistical modelling to test causal hypotheses.

## Zusammenfassung

Hörverlust ist ein Indikator des Risikos für kognitiven Abbau und Demenz. Studien über kontrollierte Hörinterventionen mit langfristigen Ergebnissen zur kognitiven Leistungsfähigkeit stellen eine Herausforderung dar, daher stammen die Beweise für die Auswirkungen von Hörbehandlungen in erster Linie aus Beobachtungsstudien und werden wahrscheinlich auch weiterhin aus anderen Studien als randomisierten kontrollierten Studien herrühren. Überprüft wurden 7 Studien zu Hörbehandlungen mit Ergebnissen zur kognitiven Leistungsfähigkeit, die über einen Zeitraum von mehr als 3 Jahren durchgeführt wurden. Die meisten waren von geringer bis mittlerer Qualität. Eine Studie zu Cochleaimplantaten führte zu unklaren Ergebnissen. Von 6 Hörgerätestudien wurde bei dreien über eine positive Auswirkung der Nutzung von Hörgeräten berichtet, während in den anderen 3 Studien keine Auswirkung der Nutzung von Hörgeräten auf den kognitiven Abbau oder auf eine auftretende kognitive Beeinträchtigung festgestellt wurde. Weitere Studien sind erforderlich, um den Nutzen von Hörbehandlungen für die langfristigen Ergebnisse in Bezug auf die kognitive Leistungsfähigkeit zu verdeutlichen. Wissenschaftliche Untersuchungen sollten objektiv ermittelte Hördaten, theoretisch begründete Ergebnisse zur kognitiven Leistungsfähigkeit einschließlich Demenz-Subtypen, Charakterisierung und Berücksichtigung von Störfaktoren und die Anwendung hochentwickelter statistischer Modelle bei der Prüfung kausaler Hypothesen umfassen.

Identifying treatments and management strategies to prevent or delay the onset of dementia is an international priority. A recent review of modifiable risk factors for dementia concluded that up to 35% of dementia cases may be preventable. The review also identified that around 9% of dementia cases may be attributable to hearing loss in midlife.

The proportion of potentially preventable cases attributable to hearing loss at a population level was relatively high partly because hearing loss is highly prevalent among those aged over 50 years [1]; in the United Kingdom, 29% of adults aged 55–74 years have a hearing impairment (pure-tone audiometric thresholds >25 dB hearing loss [HL] in the better ear; [2]).

The conclusion that 9% of dementia cases are linked to hearing loss was based on a meta-analysis of three studies that linked baseline levels of hearing loss to risk of incident dementia [3–5]. The limitation of these observational studies was that although they were able to show that hearing loss is a marker of dementia risk, they were not able to establish causation. Alternative explanations to hearing loss contributing causally to dementia risk include (a) hearing loss and dementia being related owing to common causes, (b) cognitive factors impacting on hearing or (c) cognitive decline impacting on hearing [6, 7]. For example, hearing loss may be a marker of neurological frailty due to processes that impact on both hearing and cognition; cognitive decline/dementia and hearing loss share numerous genetic, medical, social, economic and health behavioural factors [8, 9]. Alternatively, cognitive factors may impact the performance in hearing tests [10], and cognitive difficulties increase the likelihood of ‘hearing difficulties’ being reported [11]. Hearing could plausibly impact on cognition, either directly by alterations in auditory input impacting on brain structures that support cognition [12] or indirectly via social isolation, depression, reduced self-efficacy, reduced engagement in cognitively stimulating activities or reduced physical activity [13].

Hearing loss may be a marker of neurological frailty

A key question, then, is whether hearing loss is causally linked to the risk of cognitive decline and dementia and whether treating hearing impairment (e. g. with hearing aids) may reduce cognitive decline and risk of dementia.

Some hearing aid and cochlear implant intervention studies have measured cognitive outcomes; the results are mixed [14–18]. But the main limitation of these studies was their short duration; cognitive decline is gradual [19], and studies need to be at least several years in duration to be able to observe the effects of hearing interventions on cognitive decline. Controlled trials of hearing interventions with cognitive outcomes measured over several years would be desirable.

However, well-controlled hearing intervention studies with long-term cognitive outcomes are challenging. In addition to the long durations required to examine cognitive decline, studies interested in incident dementia would need to be large in size as well as long in duration (incidence of dementia increases rapidly with age, but is only around 8 cases per 1000 person-years for those aged 70–74 [20]). Such large and long-running studies would be very expensive. There are also challenges with longitudinal designs. Longitudinal studies of cognition are subject to problematic practice effects on cognitive outcome measures and selective drop-out, which biases results [21].

Controlled trials of hearing interventions are challenging

Adherence to some hearing interventions may be problematic. Up to 30% of hearing aid owners do not use their hearing aids or use them infrequently (<4 h/day; [22]), and thus hearing aid intervention studies must take non-adherence into account in sample size calculations. Finally, there are ethical considerations for hearing intervention studies. Ideally, an intervention study would utilise a randomised controlled design. One would randomly allocate people identified with a hearing impairment to a hearing intervention or control condition and measure long-term cognitive outcomes for both groups. But because the benefits of hearing aids and other hearing interventions are well established [23], it is ethically problematic to withhold them from the people in a control group while providing them to those in the intervention group, particularly over the long study durations that would be required.

Some researchers have grappled with these issues, and prospective studies of hearing interventions for long-term cognitive outcomes are planned or under way [24–27]. However, the limitations of conducting well-controlled hearing intervention studies with long-term cognitive outcomes means that the primary source of evidence will come from studies other than randomised controlled trials.

## Overview of hearing intervention studies

The following is an overview of studies of hearing interventions for adults with normal cognition that evaluated cognitive outcomes (including cognitive change and incident cognitive impairment) over durations longer than 3 years that were published up to December 2018 (and that the author is aware of; Table 1). Studies with a minimum follow-up of 3 years were chosen because it is conceivable that cognitive decline may be observable during timescales of at least 3 years [28]. The level of evidence of all the studies was low to moderate [29].

**Table 1:** Hearing intervention studies^a^

Study	Intervention	Study design^b^	Participants	Outcome measures	Results
Mosnier et al. 2018 [30]	Cochlear implant	Level 4 (uncontrolled single-group study)	Cochlear implant recipients (*n* = 70; including *n* = 30 with MCI) aged >65 (average age 72 at baseline)	Cognitive test battery (MMSE, 5‑word test, clock drawing, d2 Test of Attention, Trail Making, Fluency) at 1 year and 7 years post-implantation	*Cognitive test battery* Worse performance (MMSE, Clock Draw, d2 Test of Attention, TMT, Fluency letters); no difference (5-word test, Fluency categories) between 1 and 7 years post-implant
				Dementia status, determined by a physician on the basis of (a) significant impairment in social and activities of daily living and (b) worse performance on two or more tests from the cognitive test battery at 7 years vs 1 year	*Dementia/MCI* Of 31 participants with MCI at baseline, two developed dementia
				MCI, greater degree of cognitive decline ‘than expected for age’ on the cognitive test battery without impairment in daily functioning	Of 38 participants with normal cognition, 12 developed MCI
Maharani et al. 2018 [31]	Hearing aid	Level 3 (non-randomised controlled cohort)	Adults over 50 who began using a hearing aid between 1996 and 2014	Episodic memory scores assessed every 2 years (between 6 and 9 yearsʼ follow-up)	Reduction in rate of decline in memory following commencement of hearing aid use (β = −0.02 vs −0.10; adjusted for age, sex, education, marital status, smoking, alcohol consumption, physical activity, depression score and health comorbidities)
Amieva et al. 2015 [32]	Hearing aid	Level 3 (non-randomised controlled cohort)	150 hearing aid users; 1126 adults self-reported HL; 2394 adults with self-reported NH, all aged >65 years at baseline	MMSE, approximately every three years for 25 years	Slower rate of decline for hearing aid users vs NH (difference in rate of decline: β = 0.05; adjusted for age, sex, educational level, depression, living situation, social network, comorbidities, medication, dementia)
Deal et al. 2015 [33]	Hearing aid	Level 3 (non-randomised controlled cohort)	Adults with moderate/severe hearing impairment (better ear >40 dB HL); 42 non-hearing aid users and 43 hearing aid users	Three cognitive tests in 1990–92, 1996–98 and 2013; Memory (Delayed Word Recall), language (Word Fluency), processing speed/attention (Digit Symbol Substitution Test)	Slower decline for hearing aid users vs non-users (β = −1.45 vs −0.97) on cognitive composite score (adjusted for age, age^2^, sex, education, smoking status, diabetes, hypertension, and Wide Range Achievement Test score)
Dawes et al. 2015 [34]	Hearing aid	Level 3 (non-randomised controlled cohort)	Adults with hearing impairment (better ear >40 dB HL); 597 non-hearing aid users and 69 hearing aid users, average age 68 years at baseline	MMSE at 5 and 11 years post-baseline; Trial Making, Auditory Verbal Learning Test, Digit Symbol Substitution Test, Verbal Fluency at 11 years post-baseline	No differences between hearing aid users and non-users in cognitive tests or incidence of cognitive impairment at any time point (adjusted for age, sex and hearing level)
				Incident cognitive impairment (reported diagnosis or MMSE <24)	
Lin et al. 2013 [35]	Hearing aid	Level 3 (non-randomised controlled cohort)	Adults with hearing impairment (better ear 40–70 dB HL; based on audiometric evaluation at year 5 of a 6-year follow-up); 218 non-hearing aid users and 182 hearing aid users, average at 78 at baseline	3MS (global function) and Digit Symbol Substitution (processing speed/attention) 6 years post-baseline	No differences between hearing aid users and non-users in rate of decline or incidence of cognitive impairment (adjusted for age, sex, ethnicity, education, study site, smoking, hypertension, diabetes, and stroke history)
				Incident cognitive impairment (3MS score <80 or decline in 3MS >5 points from baseline)	
Valentijn et al. 2005 [36]	Hearing aid	Level 3 (non-randomised controlled cohort)	391 adults with mean hearing level 16 dB HL (range 0 to 58) at baseline, including 7 who obtained a hearing aid between baseline and follow-up	MMSE, Visual Verbal Learning Test, Stroop Colour Word Test, Concept Shift Test, Verbal Fluency, Letter–Digit Substitution at baseline and 6‑year follow-up	No interaction with hearing aid use and cognitive change at 6‑year follow-up (adjusted for age, sex, and education)

*HL* hearing loss, *MCI* mild cognitive impairment, *MMSE* Mini-Mental State Examination, *NH* normal hearing

^a^Studies of adults with normal cognition with cognitive outcomes assessed over longer than 12 months

^b^According to the *Oxford 2011 Levels of Evidence *(OCEBM Levels of Evidence Working Group, 2011): Level 1, fully powered randomised controlled trials or meta-analysis; Level 2, controlled trials without randomisation; Level 3, retrospective cohort or case-control studies; Level 4, case series or uncontrolled single-group study; and Level 5, expert opinion or case report

### Cochlear implants

One study concerned cochlear implants [30]. Cochlear implant interventions may be of particular interest because cochlear implants have a more dramatic impact on hearing function than hearing aids do. Cochlear implants restore functional hearing to someone with profound hearing loss, while hearing aids provide an incremental increase in audibility of sounds that were already partially audible prior to using a hearing aid. One might therefore predict that the impact of cochlear implant interventions on cognitive outcomes may be more substantial than that of hearing aid interventions.

Mosnier and colleagues [30] tested 70 cochlear implant recipients at 1 and 7 years after implantation. The sample included 31 people who were identified as having mild cognitive impairment (MCI) at baseline. The remaining participants had normal cognitive function. This study was included in this review because incident cognitive impairment data (but unfortunately not cognitive test performance data) were reported separately for those with normal cognition versus MCI. There were significant declines in performance in five out of seven cognitive tests, with no change in the remaining two tests (for the whole sample, including those with MCI and normal cognition). Mosnier et al. reported rates of incident MCI and dementia, and concluded that these rates were lower in both the MCI and normal cognition groups than might have been expected based on population data for incidence of cognitive impairment (although this comparison was not tested statistically, and it was unclear why one might expect incidence of cognitive impairment to be *lower* among cochlear implant users than among the general population of people with mostly normal hearing). A further limitation of this analysis was that the diagnosis of MCI/dementia was based partly on the study cognitive tests, which were repeatedly administered and were likely to be subject to practice effects. The lack of a control group makes it difficult to isolate the impact of cochlear implantation on the cognitive outcomes reported in this study.

### Hearing aids

Studies modelling the impact of hearing aid use on cognitive outcomes have mixed findings. Some studies reported a reduction in the rate of cognitive decline associated with hearing aid use [31–33], while others reported no association with hearing aid use and the rate of cognitive decline [34–36] or incidence of cognitive impairment [34, 35].

Hearing measures have tended to be neglected in large epidemiological data resources, perhaps owing to the lack of recognition of hearing loss being an important public health issue and/or the difficulty in assessing hearing using gold standard audiometric methods in large-scale studies. However, most of the aforementioned studies utilised large data sets that contained gold standard audiometric measures of hearing.

### Cognitive outcome measures

Studies used a variety of cognitive outcome measures, which may explain some variation in findings. Studies included tests of a range of cognitive domains including attention, processing speed, executive functioning, short-term memory, working memory and long-term memory. Some used screening tests for cognitive impairment such as the Mini Mental Status Exam [37] that may be relatively insensitive to age-related cognitive change. Most studies did not provide a strong justification for the choice of cognitive outcome measures. The choice of outcome may have been primarily a pragmatic one, based on what data were available. Some researchers posit different effects of hearing loss on different cognitive domains ([38, 39]; although a recent meta-analysis concluded that the correlation with hearing impairment was similar across cognitive domains [16]). Future research should offer a rationale for the chosen cognitive assessments based on a hypothesised mechanism of action and/or clinical or functional relevance.

### Dementia subtypes

With respect to dementia outcomes, all research to date concerning hearing loss and risk of dementia has made no differentiation according to type of cognitive pathology and focused on ‘all-cause’ dementia. Dementia is a symptomatic description of cognitive impairment that impacts on functioning in daily life.

There are many types of dementia with different pathological causes

But there are many types of dementia with different pathological causes. Future research should differentiate between dementia types and test specific hypotheses concerning how hearing impairment may contribute causally to specific dementias. Lack of information about dementia subtype and/or small numbers of people with subtypes of dementia in existing data sets is a limitation. Nevertheless, availability of routinely collected clinical data for large numbers of people (e.g. the UK National Institute of Health Research’s Health Informatics Collaborative, http://www.hic.nihr.ac.uk/) including information on dementia subtypes and hearing may facilitate this sort of research in future.

### Demographic confounds

One important caveat for observational studies of hearing aid use such as those described here is that hearing aid users are an elite group with respect to demographics, health and cognitive outcomes. Only around 10–20% of people with hearing loss use hearing aids, and hearing aid users tend to be more affluent, better educated and more likely to be members of majority ethnic groups than non-hearing aid users [40–42]. Cochlear implant users may also differ from the general population in important ways [43]. These demographic factors are associated with a range of health outcomes including cognitive decline and dementia (e. g. [44, 45]). The observational studies discussed here attempted to minimise demographic and health confounds by measuring and adjusting for them statistically. The issue of hearing aid users being an elite group may partly explain why Amieva et al. [32] reported a lower rate of cognitive decline for hearing aid users compared with those who reported *normal hearing*. The analysis of Ameiva et al. did not compare cognitive decline between hearing aid users and non-users among those with reported hearing impairment or provide demographic details about the small sample of hearing aid users in the study. It is important to characterise hearing aid users as thoroughly as possible and statistically control for demographic differences that may impact on cognitive outcomes.

Other studies have dealt with the issue of bias in samples of hearing aid users in other ways; Dawes et al. [13] utilised a sample that was relatively demographically homogeneous (from a small predominantly ethnically white town in rural United States), where there were no demographic differences between hearing aid users and non-users. Alternatively, Maharani et al. [31] examined differences in rates of cognitive change before and after hearing aid use within the same individuals.

## Future directions

Intervention studies are required to elucidate the significance of hearing loss as a marker of risk for cognitive decline and dementia, and to inform public health efforts to reduce the incidence and prevalence of dementia. But well-controlled prospective intervention studies involve significant practical, scientific and ethical challenges. Observational studies will continue to be a major source of evidence. Large data sets should include measures of hearing and hearing interventions in addition to longitudinal cognitive outcomes. Access and linkage to routinely collected clinical audiological and cognitive data would also facilitate insight into links between hearing loss and cognitive outcomes, as well as the benefit of hearing interventions. Application of structural equation modelling methodology with longitudinal data would enable testing of specific hypotheses for the relationship between hearing loss and cognitive decline and the mechanisms of effect of hearing interventions on cognition (e. g. [13]), which could then inform prospective intervention studies.

If hearing loss is causally implicated in dementia risk, then effective prevention, identification and treatment of hearing loss may be critically important to reduce the cumulative incidence of dementia.

## Practical conclusion

- Hearing loss is a marker of risk for cognitive decline and dementia.
- Controlled hearing intervention studies of long-term cognitive outcomes are challenging, and thus the evidence for the impact of hearing interventions comes from observational studies.
- Further studies are needed to assess the benefit of hearing interventions on long-term cognitive outcomes and to guide public health efforts to lower the incidence and prevalence of dementia.
